# The Relationship Between Demographics, Behavioral and Experiential Engagement Factors, and the Use of Artistic Creative Activities to Regulate Emotions

**DOI:** 10.1037/aca0000296

**Published:** 2020-01-13

**Authors:** Daisy Fancourt, Claire Garnett, Daniel Müllensiefen

**Affiliations:** 1 Department of Behavioural Science and Health, University College London; 2 Department of Psychology, Goldsmiths, University of London

**Keywords:** creativity, arts, emotion regulation, engagement, emotions

## Abstract

There has been increasing interest in the role of artistic creative activities in supporting emotion regulation. However, there is little research about how demographic factors (such as age, gender, ethnicity, personality, and socioeconomic status) or factors relating to creative engagement (including engagement behaviors and subjective experience of engagement) influence our ability to use artistic creative activities to regulate our emotions. We analyzed data from 40,949 adults and used a structural equation modeling approach to model the relationships among demographic factors, factors relating to engagement, and our use of emotion regulation strategies (ERSs) while engaging in artistic creative activities. We found that women make more use of creative activities to regulate their emotions than do men, as do those of lower socioeconomic status. Training in doing an artistic activity, regular engagement, and enjoyment while engaging are all associated with a greater ability to use artistic activities to regulate our emotions. We also identified relationships between demographic and engagement factors and specific types of ERSs, such as avoidance strategies (e.g., distraction, suppression, or detachment from negative or stressful emotions), approach strategies (e.g., acceptance, reappraisal and problem solving), and self-development strategies (e.g., enhanced self-identity, improved self-esteem, and increased agency). Artistic creative activities are increasingly being recognized as effective ways of regulating emotional responses. Overall, this study provides insight into the interrelationship between individual attributes, modifiable patterns of engagement and emotion regulation when engaging in artistic activities.

Over the last 25 years there has been increasing research interest in emotion regulation. This work has emerged from broader literature on coping (Lazarus & Folkman, 1984), but is distinguished through its focus on shorter periods of time (e.g., the regulation of emotions in immediate response to daily events or experiences as opposed to longer term coping such as with bereavement; Gross, 2015). This ability to self-regulate emotions is critical in allowing individuals to adjust emotional responses to meet situational demands (Gross & Thompson, 2007). Cumulatively, the effective implementation of emotion regulation strategies (ERSs) has been consistently linked with greater well-being, lower levels of mental illness, and greater satisfaction with life (Aldao, Nolen-Hoeksema, & Schweizer, 2010; Gross & John, 2003).

In parallel, there has been development in literature on the impact of artistic creative activities (such as performing arts, visual arts, literature, online digital and electronic arts, and cultural engagement) on well-being, mental health, and life satisfaction (Daykin et al., 2018; Fujiwara & MacKerron, 2015; Mansfield et al., 2018; Tischler, 2010), with emotion regulation identified as a key mechanism by which these wider benefits are realized (Fancourt, Garnett, Spiro, West, & Müllensiefen, 2019). However, although there is a substantial literature on factors that affect both our use of ERSs and our engagement in artistic creative activities including demographic, socioeconomic, and behavioral factors, much less is known about how such factors are related to our ability to use artistic creative activities to regulate our emotions.

## Use of ERSs

There are potentially limitless numbers of ERSs (Gross, 2001): For example, concentration on the emotion (also called *rumination*) involves the repetitive direction of our attention to our feelings or their consequences; *distraction* involves focusing our attention away from the situation or our feelings toward it; *reflection* or *reappraisal* involves changing a situation’s meaning in a way that alters its emotional impact; *problem solving* involves specifically addressing a situation to try and resolve it and thereby resolve the emotions associated with it; *suppression* involves trying to squash negative emotions; and *discharge* involves attempting to release or vent negative emotions (Gross & Thompson, 2007). Although some literature suggests that certain strategies are more adaptive than others (Aldao et al., 2010), the way these strategies are implemented within specific contexts is also key for good mental health (Kashdan et al., 2014). However, what remains less well understood is how different factors affect our individual ability to apply these strategies in different contexts.

Broadly, there has been a call for more research focused on understanding the role of demographic factors on the use of ERSs (Aldao, 2013). However, research to date is contradictory. For example, studies involving nonclinical samples have found gender differences in emotion regulation, with women typically using more ERSs simultaneously and having to make more effort in using cognitive regulation strategies such as reappraisal (McRae, Misra, Prasad, Pereira, & Gross, 2012; Nolen-Hoeksema & Aldao, 2011). Other studies have suggested that men and women in fact differ in the type of ERSs most commonly used, with women making more use of rumination but less use of avoidance and suppression strategies (Zimmermann & Iwanski, 2014). Similarly, although some studies have shown a negative relationship between age and use of ERSs (Nolen-Hoeksema & Aldao, 2011), others have found that use of strategies increases with age (Zimmermann & Iwanski, 2014). Similarly, socioeconomic status may moderate the use and success of ERSs. In particular, studies have repeatedly demonstrated the negative effects of low socioeconomic status (SES) on emotional development (Raver, 2004). However, the effects of this on emotion regulation are mixed. At the extreme, poverty has been linked with lower levels of competent emotional self-regulation (Evans & English, 2002; Lengua, 2002), and higher levels of disposable income and SES have been associated with implementation of ERSs (Côté, Gyurak, & Levenson, 2010). Yet other studies have shown that because lower SES affords an individual less control over their environment, the use of strategies to regulate emotions is more beneficial than for people from higher SES backgrounds (Troy, Ford, McRae, Zarolia, & Mauss, 2017). Research has also shown how other demographic factors, such as open personality type, are linked with greater use of ERSs (Mayer et al., 2004).

## Use of ERSs When Undertaking Artistic Creative Activities

Although emotion regulation is continuous and can be automatic across our daily lives, it can also be explicitly undertaken either in the face of emotional situations or through engagement in specific activities (Gyurak, Gross, & Etkin, 2011). In recent years, there has been increasing interest in the role of artistic creative activities in supporting emotion regulation. Artistic creative activities can refer to “Big C” activities (i.e., the remarkable creative achievements of a select few) or “little c” activities (i.e., engagement in everyday creative artistic activities; John-Steiner, 2015). These little c activities are commonly categorized into different types: performing arts (such as singing, dancing, and acting); visual arts, design, and craft (such as sewing, painting, and woodwork); literature (such as reading and creative writing); online digital and electronic arts (such as photography, filmmaking, and digital graphics design); and community and cultural festivals, fairs, and events (Davies et al., 2012). A number of studies have explored the link between creative activities, moods and emotions, leading to the proposition that bidirectional pathways link creative activities with emotions (Baas, De Dreu, & Nijstad, 2008; Dreu, van der Wilk, Poppe, Kwakkel, & van Wegen, 2012; Ivcevic & Brackett, 2015). However, there is less research on how creative activities affect emotion *regulation*.

Creative activities recruit strong cognitive processes for supporting emotion regulation. They involve cognitive flexibility, such as adopting multiple perspectives (which can help in ERSs, such as reappraisal), considering novel solutions (which can help in ERSs, such as problem solving), and achieving new relationships to situations (which can support a number of ERSs, such as acceptance; De Dreu, Baas, & Nijstad, 2008; Lubart, 2001). They can also provide a mindful space, which supports distraction (Chiesa, Serretti, & Jakobsen, 2013), and can support in catharsis as a way of regulating negative emotions (Bushman, Baumeister, & Phillips, 2001). In line with this, studies on artistic creative activities have shown there are three broad categories of emotion regulation when engaging in these activities: strategies that involve avoiding stresses or negative situations (e.g., distraction, suppression or detachment from negative or stressful emotions), strategies that actually involve addressing these stresses or situations (e.g., acceptance, reappraisal and problem solving), and strategies that help us develop ourselves to cope with such stresses or situations more effectively (e.g., enhanced self-identity, improved self-esteem and increased agency; Fancourt et al., 2019). Experimental studies have explored some of these strategies in more detail, including studies showing the role of discharge (catharsis), acceptance, and distraction when drawing (Dalebroux, Goldstein, & Winner, 2008; De Petrillo & Winner, 2005; Drake, Coleman, & Winner, 2011; James, Drake, & Winner, 2018), and of reappraisal and acceptance when engaging in creative writing (Pennebaker, Mayne, & Francis, 1997). But the potential relationships between demographic factors, factors relating to creative engagement and use of ERSs have only been explored in a very limited way.

Among previous literature, a few studies in music listening have shown gender differences for certain ERSs such as discharge (a process of releasing or venting emotions) and diversion (refocusing thoughts onto different foci; Carlson et al., 2015). Other studies have found that although the same ERSs appear to be used across the life span, individuals report becoming better at employing these ERSs as they age (Saarikallio, 2011). However, these studies have involved listening to music rather than actively engaging in an artistic creative activity, so the potential relationships between demographic factors and use of ERSs when undertaking creative activities remains to be explored further. Nevertheless, alongside the literature on the relationship between demographic factors and emotion regulation, there is a large literature showing how engagement with the arts is related to demographic factors. For example, women and people who are younger are more likely to engage, as are those of higher social class and higher educational attainment and those with open personality types (McManus & Furnham, 2006). Consequently, the relationship between demographic factors and use of ERSs when engaging in an artistic creative activity was the first point of focus for this study. Building on the preliminary findings described above suggesting demographic differences in use of ERSs generally (outside of creative activities), our first aim was to explore if core demographic factors (e.g., age, gender, ethnicity, and SES) are related to the use of ERSs when engaging in creative activities.

Relatedly, there is also very little research exploring how other factors relating to creative engagement affect ERSs, so this formed our second point of focus for this study. *Engagement* is frequently defined as consisting of behaviors and subjective experience (Perski, Blandford, West, & Michie, 2017). In relation to behaviors, some previous studies of specific creative activities have shown no association between frequency of engagement or experience and emotional responses (Fancourt & Williamon, 2016), but there is little research about engagement and emotion regulation. It could be hypothesized that experience, training and frequency of engagement would be associated with greater use of ERSs given the large body of research showing that these factors can be used to train individuals with music performance anxiety to better regulate their anxiety (Kenny, 2005). Indeed, studies have suggested that experience enables individuals to make more targeted use of listening to music to help them regulate their emotions (Saarikallio, 2011). But equally, curiosity has been identified as a positive motivational component that is associated with self-regulation (Kashdan, Rose, & Fincham, 2004), which could suggest that engaging in creative activities that an individual has not engaged with before (*novel activities*) could have a greater effect on ERSs, and therefore factors related to past experience may not significantly affect our use of ERSs. So, our second aim was to explore whether behavioral aspects of engagement are related to the use of ERSs when engaging in creative activities.

Finally, it also remains unknown whether aspects of subjective experience relating to engagement affect the use of ERSs when engaging in creative activities. So, for our third aim, we explored whether enjoying an activity is related to the use of ERSs, and also whether perceiving that an activity is helping with emotion regulation is related to engagement with that activity and the process of emotion regulation. We also explored whether perceiving oneself to be good at an activity (perceived talent) is related to both subjective experience of engagement and behaviors relating to engagement.

**Figure 1 fig1:**
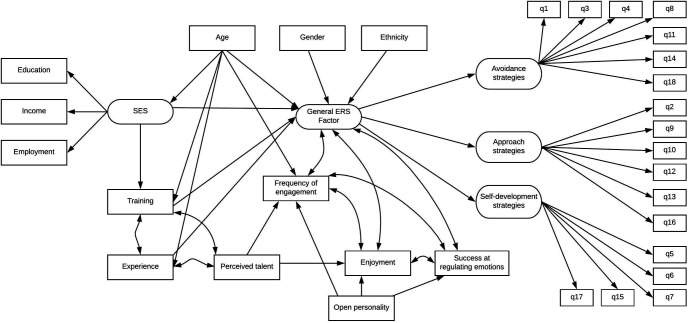
Hypothetical model linking demographic factors, factors relating to creative engagement, and use of emotion regulation strategies (ERSs).

Consequently, this study explored whether individual demographic factors and factors relating to creative engagement (both behavioral and experiential) are related to the use of ERSs when engaging in artistic creative activities (see Figure 1). Specifically, we examined the role of demographic factors (including age, gender, ethnicity, personality and socioeconomic status) and factors relating to creative engagement (including experience, training, frequency of engagement, enjoyment, self-rated talent, and perceived success at regulating emotions). As this study focuses on a number of interrelated factors, we used a structural equation modeling approach involving a large sample of adults that allows us to simultaneously model the relationships between all included variables.[Fig fig1]

## Method

### Procedure

We used data from the BBC Great British Creativity Test, which is a cross-sectional data set gathered across 11 weeks in 2018 from 47,924 people aged 18 or over in the United Kingdom. Although not a representative sample, the data set exhibits good distribution across core demographic and socioeconomic factors (Fancourt et al., 2019). We excluded participants who reported never engaging in any of the artistic creative activities we were focusing on (*n* = 793) and participants who did not wish to disclose their gender (*n* = 459) or income (*n* = 6,030). This provided a final analytical sample of 40,949.

Participants were 55.1% (*n* = 22,563) female, with an average age of 47.0 years (*SD* = 14.3, range = 18–93), majority White British or Irish (91.4%). Participants were invited to provide demographic data as well as select their preferred creative activity and answer questions on their engagement with that activity and the ERSs they typically used while engaging with it. Although this study focused on creative activities in general rather than on specific activities, details on preferred activities are provided along with demographics in Table 1. The original study was approved by University College London Research Ethics Committee (Reference 12467/001), and all participants gave informed consent to data collection and use of the data in subsequent analyses.[Table tbl1]

### Measures

*Artistic creative activities* were defined in the dataset following a theorized model for population-level research (Davies et al., 2012) and as consisting of performing arts (singing; dancing; playing a musical instrument; rehearsing or performing in a play/drama/opera; learning or practicing magic tricks or circus skills); visual arts, design, and craft (painting, drawing, printmaking, sculpture, pottery, calligraphy, or jewelry making; textile crafts e.g., embroidery, crocheting, or knitting or wood crafts, such as carving or furniture making), literature-related activities (reading a novel, stories, poetry or plays for pleasure; creative writing; and composing music), and online digital and electronic arts (creating artworks or animations on a computer, making films or videos, photography). As we focused specifically on active participation in creative activities for this study, we did not include engagement in community and cultural festivals, fairs, and events, as these constitute receptive engagement (Davies et al., 2012). We followed the theoretical standpoint that artistic creative activities are multimodal activities (Craig et al., 2008). Although each artistic activity might have distinct properties different from other artistic activities, all artistic activities involve consistent underlying components that are inherent to them being artistic, such as the use of imagination, cognitive stimulation, experiential pleasure, sensory activation, and the cultivation of individual skills (Dutton, 2006). According to this theoretical approach, the major distinguishing feature between different artistic creative activities is personal preference. Therefore, we asked participants to focus on the creative activity they felt was most effective at regulating their emotions for this study when answering the study questions.

ERSs were measured through self-report of usual spontaneous usage of different strategies when engaging in creative activities of choice rather than through experimental manipulation. Specifically, we used the Emotion Regulation Strategies for Artistic Creative Activities (ERS-ACA) scale (Fancourt et al., 2019). The ERS-ACA scale is a validated 18-item measure that includes an overall factor for use of ERSs when engaging in artistic creative activities as well as three subscales of strategies: (1) avoidance, (2) approach, and (3) self-development. *Avoidance strategies* (e.g., distraction, suppression or detachment from negative or stressful emotions) are measured with questions such as “I can block out any unwanted thoughts or feelings,” “I can shake off any anxieties in my life,” and “it helps me to disengage from things that are bothering me.” *Approach strategies* (e.g., acceptance, reappraisal, and problem solving) are measured with questions such as “it helps me refocus on what matter in my life,” “it helps me to come to terms with my own emotions,” and “it helps me to understand my own feelings on things that are on my mind.” *Self-development strategies* (e.g., enhanced self-identity, improved self-esteem, and increased agency) are measured with questions such as “I feel more confident in myself,” “it boosts my self-esteem,” and “it gives me a sense of purpose.” The scale has previously been validated and has shown good psychometric properties including strong internal reliability (overall factor α = .93, avoidance strategies factor α = .9, approach strategies factor α = .88, self-development strategies factor α = .88), good convergent and divergent validity with previous scales, and strong test–retest validity (Pearson’s *r* = .85, *p* < .001; Spearman’s correlation coefficient ρ = 0.80, *p* = .001; Fancourt et al., 2019).

For demographic factors, participants self-reported their sex (reference male), age, and ethnicity (White British/Irish/other, Asian/Asian British/Bangladeshi/Indian/Pakistani/other, Black/Black British/African/Caribbean/other, Chinese/Chinese British, mixed race, other/prefer not to say; recoded into White British/Irish/other vs. other). Socioeconomic status was assessed using three variables: education (no formal qualifications, GCSE/CSE/O-levels or other age 16 attainment, A-levels or other post16 attainment, undergraduate degree, postgraduate degree), employment status (in full-time employment, in part-time employment/self-employed, in education, retired, not working; recoded as working/studying vs. not), and income (<£16,000, £16,000–£29,999, £30,000–£59,000, £60,000–£89,000, £90,000–£119,999, >£120,000). Although not a target variable in our analyses, we also included personality type in our model, as this has been shown to predict various aspects of engagement with creative activities and is linked with mental health (Kendler, Gatz, Gardner, & Pedersen, 2006; Matsudaira & Kitamura, 2006; McManus & Furnham, 2006). While not a direct confounder in the relationship between demographics or engagement and use of ERSs, it felt important to include it within the wider model. Specifically we included the personality trait of ‘openness’ drawn from the short 15-item version of the Big Five Inventory (Lang, John, Lüdtke, Schupp, & Wagner, 2011), as this personality trait has been linked most with creative engagement (McManus & Furnham, 2006).

*Engagement* is frequently defined as consisting of behaviors and subjective experience (Perski et al., 2017). In relation to behaviors, we included three factors relating to experience and three factors relating to reward. For experience, participants reported number of years doing the activity (recoded into <1 month, 1 to 12 months, 1 tol 5 years, 6 to 10 years, 11 to 20 years, 21 to 40 years, 40+ years), number of years of training in the activity (recoded into none, <1 year, 1 to 5 years, 6 to 10 years, 11+ years), and frequency of engagement with the activity (a few times a year, once or twice a month, once a week or more, daily). In relation to subjective experience, we measured self-rated enjoyment of the activity (from 1 = *not at all* to 5 = *very much*), self-rated talent in the activity (from 1 = *not at all talented* to 5 = *very talented*), and self-rated effectiveness of the activity at regulating emotions (not at all, not very, a little, quite, very).

### Construction of the Structural Equation Models

Although decisions on the causal ordering of factors within structural equation models (SEMs) are recognized as challenging (Pearl, 2000), this model was built based on the literature outlined in the preceding text and based on logical assumption of certain factors. For example, age, gender, and ethnicity are inevitably exogenous, so they were provided as influencers of other factors but not as being influenced themselves (see Figure 1). SES was considered as consisting of education, income, and occupational status, as these factors have repeatedly been used to derive SES in other studies. We considered SES as a likely driver of training and use of ERSs and considered that other aspects of engagement behaviors such as experience also were likely to predict use of ERSs. We considered that talent itself might not be related to use of ERSs but could be related to frequency of engagement and enjoyment. We considered all behavioral engagement factors and all subjective experience engagement factors likely had bidirectional relationships with one another. It is possible that further interconnections between our demographic and engagement factors could exist, but to avoid overloading the model, we focused on how these factors interacted with use of ERSs. We further provide the correlation matrix (see Table 1 in the online supplemental material) for readers to consider how the model could be reworked using different assumptions.

### Statistical Analysis

Analyses were carried out in R Version 3.5.1 using the *lavaan* package. We fitted two SEMs to determine the relationship between ERSs and various demographic and engagement variables. The first SEM included the general factor for ERSs to assess how demographic and engagement factors are associated with the broad use of ERSs, testing our three broad aims. As a sensitivity analysis, the second SEM included the three specific factors for ERSs in order to explore if there were differential associations between individual ERS factors and demographic, behavioral, and experiential variables in the model. Having confirmed multivariate normality, we used robust maximum likelihood estimation with Huber-White standard errors.

## Results

### General Use of ERSs

**Figure 2 fig2:**
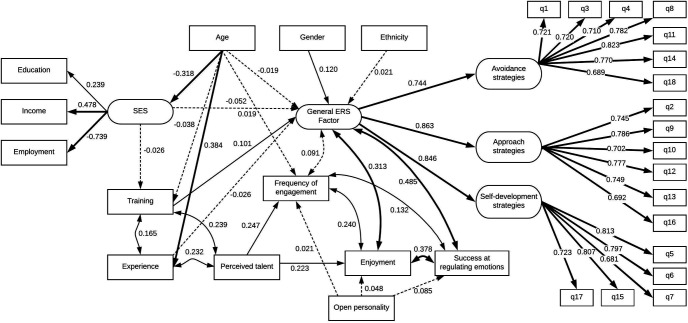
Structural equation model of demographic and activity-related factors in relation to general use of emotion regulation strategies (ERSs).

For SEM 1, all hypothesized paths were significant, and the resulting model was an acceptable fit for the data, χ^2^(416) = 55,517, *p* < .001, Tucker-Lewis Index (TLI) = 0.88, confirmatory fit index (CFI) = 0.89, root mean square error of approximation (RMSEA) = 0.057, standardized root mean squared residual (SRMR) = 0.066. The model is depicted graphically in Figure 2.[Fig fig2]

#### Demographic factors

The SEM showed that all four included demographic factors were significantly associated with the use of ERSs when undertaking creative activities. There was a very small association for age suggesting that people make less use of ERSs as they age (β = −0.019, *p* = .001), and also a very small association for ethnicity, suggesting that individuals of White British ethnicity make slightly less use of ERSs than people of other ethnicities (β = 0.021, *p* < .001). There was a slightly larger (but still very small) association showing that lower SES was also associated with greater use of ERSs (β = −0.052, *p* < .001). The only association above β = 0.1 was for gender, with evidence that women make more use of ERSs when engaging in artistic creative activities than men (β = 0.12, *p* < .001).

#### Behavioral engagement factors

Training, experience and frequency of engagement were all related to use of ERSs. People with prior training in their creative activity made more use of ERSs (β = 0.101, *p* < .001), although people who had been doing the activity for longer made less use of ERSs (β = −0.026, *p* < .001). There was also a significant covariance between frequency of engagement and use of ERSs (β = 0.091, *p* < .001).

#### Subjective experience engagement factors

Enjoyment was significantly associated with use of ERSs (β = 0.313, *p* < .001), with a larger size of association than any of the behavioral engagement factors. Similarly, perceived efficacy of ERSs at regulating emotions strongly covaried with use of ERSs (β = 0.485, *p* < .001). There was also evidence to suggest that perceiving oneself to be good at an activity is associated with increased frequency of engagement (β = 0.247, *p* < .001) and enjoyment (β = 0.223, *p* < .001) of that activity.

Overall, when considering the size of the associations, gender was the only demographic factor that showed an association above β = 0.1, training showed an association of β = 0.101, and the only factors to show an association larger than 0.2 or more with ERSs were the subjective experience engagement factors enjoyment and perceived success at using ERSs.

### Sensitivity Analysis: Use of Specific ERSs

**Figure 3 fig3:**
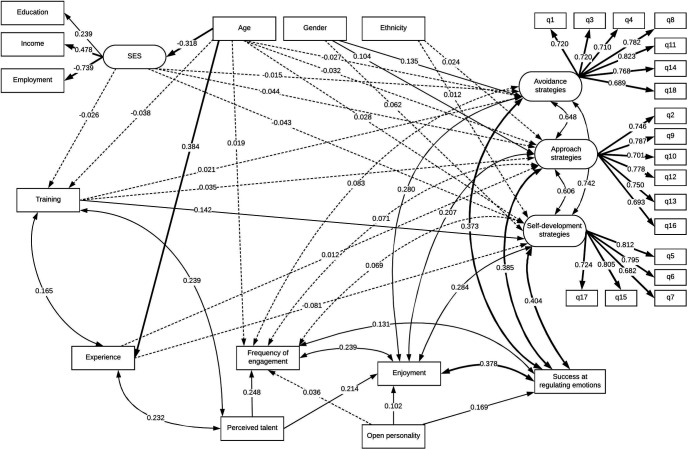
Structural equation model of demographic and activity-related factors in relation to use of specific emotion regulation strategies (ERSs).

For SEM 2 the resulting model also fitted the data well, χ^2^(395) = 52,363, *p* < .001, TLI = 0.88, CFI = 0.90, RMSEA = 0.057, SRMR = 0.059. The model is depicted graphically in Figure 3.[Fig fig3]

#### Demographic factors

When focusing on specific types of ERSs, some findings continued consistently across all three strategies. For example, women were more likely to make use of all three ERSs, and gender had a larger influence on use of avoidance strategies (β = 0.135, *p* < .001) and approach strategies (β = 0.104, *p* < .001) than self-development strategies (β = 0.062, *p* < .001). However, the size of association was still small overall. Similarly, SES was inversely related to the use of approach strategies (β = −0.044, *p* < .001) and self-development strategies (β = −0.043) as well as (but less strongly) to use of avoidance strategies (β = −0.015, *p* = .022). However, there were some differences. For example, there continued to be a small association with age, but this differed by strategy, with older adults slightly more likely to make less use of avoidance (β = −0.03, *p* < .001) and approach strategies (β = −0.03, *p* < .001) but more use of self-development strategies (β = 0.03, *p* < .001). Similarly, ethnicity was still related in a very small way to use of approach strategies (β = 0.024, *p* < .001) and self-development strategies (β = 0.012, *p* = .015), but not to avoidance strategies.

#### Behavioral engagement factors

For behavioral aspects of engagement there were also some consistencies. For example, people with prior training in the creative activity made greater use of all three strategies, in particular self-development strategies (β = 0.142, *p* < .001) but also to a lesser degrees use of approach (β = 0.035, *p* < .001) and avoidance strategies (β = 0.021, *p* < .001). However, number of years’ experience doing the activity was not associated with use of avoidance strategies, yet showed a small positive associated with use of approach strategies (β = 0.012, *p* = .032) and a small negative association with use of self-development strategies (β = −0.083, *p* < .001). Frequency of engagement, enjoyment and perceived success of the strategy at regulating emotions were consistently positively associated with use of all three ERSs with similar sizes of association for all.

#### Subjective experience engagement factors

For aspects of engagement relating to subjective experience, enjoyment continued to be positively associated with use of all three ERSs, with similar sizes of association, as did perceived success of activities in regulating emotions. Perceived talent continued to be associated both with frequency of engagement and enjoyment of the activity.

Overall, when considering the size of the associations, gender was the only demographic factor that showed an association above β = 0.1, and this was just for avoidance and approach strategies. For behavioral engagement factors, only training showed an association above β = 0.1 for self-development strategies specifically. Again, the only factors to show an association larger than 0.2 or more with ERSs were the subjective experience engagement factors enjoyment and perceived success at using ERSs.

## Discussion

Overall, this study showed that there is a relationship between demographic factors, factors relating to engagement, and our use of ERSs while engaging in artistic creative activities. Specifically, women make more use of creative activities to regulate their emotions than men, as do those of lower SES. Training in doing an artistic activity also appears to increase our ability to use it to regulate our emotions, especially our use of self-development strategies. Additionally, other factors are associated with our use of more specific types of ERSs: approach strategies are used more by those with more experience engaging in the activity; and self-development strategies are used more by those who have taken up the activity more recently. Additionally, our regularity of engaging with creative activities and our enjoyment of doing so is positively linked with our ability to use these activities to regulate our emotions.

When comparing these findings with those of previous literature, it is important to note that there are many contradictory findings about the role of demographic factors and factors relating to creative engagement on use of ERSs. However, our findings nonetheless support several previous studies. For example, in relation to demographic factors, the finding that women made more use of ERSs is in line with previous so-called ‘master stereotype’ that women are more emotional than men (Shields & Shields, 2002). Although the research literature around this theory is somewhat mixed, gender differences in emotion regulation have been shown, with women having to apply more effort in using cognitive regulation such as reappraisal (McRae et al., 2012) and also being more likely to employ avoidance strategies (Matud, 2004). Our study supports these previous results, but it is of note that the size of association we saw was very small. Therefore, although there is a difference present, this is not of a magnitude where we would expect to see major differences in the experience of creative activities, and this small difference may not actually reflect a difference in use of ERSs but just in the reporting of this use. The finding that use of avoidance and approach strategies decreased with age also supports previous literature (Nolen-Hoeksema & Aldao, 2011), although this study identified specifically that in fact self-development strategies were used more by those who were older. This could be explained by research showing that self-esteem can decline following retirement (Orth, Trzesniewski, & Robins, 2010), which is potentially the result of (at least in part) decreases in work. This could mean that creative activities that had previously been additional to work become more of a focal point and thereby have a greater impact on one’s sense of self. However, again the size of association is small, so the difference is unlikely to be large in practice. Further, the finding that those with open personality types engaged more often with ERSs when engaging in creative activities (which in turn was associated with increased use of ERSs) links in with previous literature relating creativity to open personality (Batey & Furnham, 2006), and open personality to emotion regulation (Mayer et al., 2004).

It is particularly of note that there was an inverted relationship between SES and use of ERSs. It has previously been suggested that strategies involving reappraisal are uniquely beneficial in lower SES contexts (Troy et al., 2017). Although we found a relationship between lower SES and use of all strategies, the relationship was very small for avoidance strategies and still small for approach and self-development strategies (the form of which includes reappraisal). Although there are recognized social gradients across certain arts and cultural activities (such as going to concerts or the theater; Parkinson & Buttrick, 2014), there is not such a clear social gradient across wider creative activities (Reeves, 2015). So overall, though the magnitude of association is not large, this suggests that there is certainly no deficit in the benefits that those of lower SES experience from engaging in creative activities.

It is equally notable that associations were found between various aspects of engagement and ERSs when engaging in artistic creative activities. More regular engagement with creative activities was associated with more use of ERSs. What is perhaps surprising is that, although training in a creative activity increased use of ERSs while doing that activity, the number of years’ experience doing the activity was inversely related to use of ERSs, especially in relation to self-development strategies. This supports previous research on the importance of novelty in emotional self-regulation and in enhancing self-esteem (Kashdan et al., 2004). This suggests that it is not just the engagement with creative activities that supports the regulation of our emotions, but also specifically taking part in novel creative pursuits that is important. Additionally, some of the strongest associations were found between use of ERSs and enjoyment, suggesting that affective responses to creative activities (and perhaps also well-being responses) are related to the use of these activities in regulating emotions.

This study has various strengths, including its use of a large sample, its inclusion of a validated measure of ERSs used when engaging in artistic creative activities, and its broad range of variables included within the SEM. However, as the data are cross-sectional, causality cannot be determined. Although the number of participants is large and they showed socioeconomic and demographic diversity, the sample is not nationally representative. Further, we used self-report for all variables, so for estimations of engagement, talent, and training, responses may include individual biases. Individuals vary in their self-awareness or consciousness of the use of ERSs, so this may have affected reporting, leading to a down-estimation of the use of strategies (Saarikallio, 2011). Finally, we looked at the relationship among creative activities, ERSs, and demographic factors at a single moment in time. Whether the role of demographic factors on ERSs varies over time remains unknown. Future studies may like to should consider whether the associations shown here for creative activities in general are any different for specific creative activities. These results may also guide the development of future experimental studies.

This study poses a number of future research questions. First, although we asked participants about any professional training they had, their frequency of engagement in artistic creative activities, and their self-rated talent, we did not specifically ask if they participated as professionals or amateurs. As such, it remains for future studies to explore whether use of ERSs is different when engaging in an activity for work-related or leisure-related purpose. Second, this study involved self-report of use of ERSs. Future studies could use intervention designs to test whether there are differences in use of ERSs among different populations. We also focused on a general rather than a clinical sample. Although individuals with mental illness such as depression often make less use of ERSs when engaging in activities than individuals without depression, they make very similar use of ERSs when engaging in creative activities (Fancourt & Ali, 2019). Future studies could explore the role of the demographic and engagement factors in this study in relation to more specific clinical samples to identify if we find different patterns of association. There is also a clear extension from this work to exploring the same research question among children. Previous studies have shown repeatedly how creative activities can support behaviors in children, both in terms of behavioral adjustment and classroom and learning behaviors (Fancourt & Steptoe, 2019; Kawase, Ogawa, Obata, & Hirano, 2018; Schellenberg, Corrigall, Dys, & Malti, 2015; Williams, Barrett, Welch, Abad, & Broughton, 2015). Understanding more about the ERSs that underlie these behavioral benefits and specifically whether certain children are more likely to benefit than others could have important implications for school arts classes. Finally, this study involved a sample based in the United Kingdom. Given there are different patterns of artistic and creative engagement and different responses to this engagement in different cultures (Gregory & Varney, 1996; Lamont & Thévenot, 2000), future studies could consider the potential cross-cultural validity of these findings.

These findings have several implications for practice. Emotion regulation is increasingly considered a central component of mental health, influencing a number of mental health conditions (Mennin & Farach, 2007). Artistic creative activities are increasingly being recognized as effective ways of regulating emotional responses. This research therefore supports the development of interventions that use creative activities to support individuals with mental health conditions. Further, understanding more about the interrelationship between individual attributes and patterns of engagement and how this affects emotion regulation when engaging in artistic activities is important to help identify who could benefit most from engaging in creative activities and support arts practitioners in designing and delivering effective creative activities. These findings also highlight the potential of the arts to support individuals in managing their behaviors in relation to emotions such as anger. It is well evidenced that creative activities have behavioral benefits including enhancing social cohesion and reducing aggressive behaviors (Bang, 2016; Boer & Abubakar, 2014; Moody & Phinney, 2012; Welch, Himonides, Saunders, Papageorgi, & Sarazin, 2014). Understanding the role of ERSs underlying this, and in particular how factors such as SES and past training only play a small role in the use of ERSs when engaging in creative activities, provides important detail on mechanisms and suggests the importance of creative activities when trying to support or improve emotion-related behaviors. Finally, given the finding that there is no greater benefit for individuals of higher SES (and there may in fact be benefits for those of lower SES), there could be a value to developing more targeted interventions as an effective way of supporting emotion regulation among those of lower SES experiencing stressful or negative life events who may have reduced access to such activities themselves.

In conclusion, it has previously been shown that creative activities affect our emotions via a number of ERSs. This study shows that use of ERSs when engaging in creative activities is related to a range of individual factors (including age, gender, SES, and personality) and a range of factors relating to our engagement with creative activities (including training, experience, and enjoyment).

## Supplementary Material

10.1037/aca0000296.supp

## Figures and Tables

**Table 1 tbl1:** Demographic and Creative Engagement Factors of Participants

Demographic characteristic	*N* = 40,949	Engagement	*N* = 40,949
Sex (female)	55.1%	Number of years doing the activity	
Age, *M* (*SD*)	47.0 (14.3)	Started in the last month	.5%
Ethnicity		<1 year	2.4%
White British/Irish/other	91.4%	1–5 years	11.4%
Asian/Asian British/Bangladeshi/Indian/Pakistani/other	2.6%	6–10 years	10.2%
Black/Black British/African/Caribbean/other	.7%	11–20 years	17.7%
Chinese/Chinese British	.6%	21–40 years	32.1%
Mixed race	1.4%	41+ years	25.9%
Other/prefer not to say	3.3%	Number of years of training	
Educational attainment, %		None	65.9%
GCSE/CSE/O-levels or other age 16 attainment	9.2%	<1 year	10.4%
A-levels or other post-16 attainment	15.3%	1–5 years	14.2%
Undergraduate degree	46.0%	6–10 years	4.8%
Postgraduate degree	29.5%	11+ years	4.7%
Occupational status, %		Frequency of engagement	
In full-time employment	49.4%	A few times a year	9.6%
In part-time employment/self-employed	27.6%	Once or twice a month	13.5%
In education	3.7%	Once a week or more	35.2%
Retired	15.2%	Daily	41.7%
Not working	4.1%	Self-rated enjoyment of doing the activity	
Household income		1 (*not at all*)	.2%
£16,000	11.9%	2	.8%
£16,000–£29,999	21.0%	3	6.5%
£30,000–£59,000	35.2%	4	24.0%
£60,000–£89,000	17.4%	5 (*very much*)	68.5%
£90,000–£119,999	7.8%	Self-rated talent in the activity	
>£120,000	6.8%	1 (*not at all talented*)	6.8%
Creative Activity		2	11.5%
Preferred activity		3	39.6%
Singing	12.4%	4	29.4%
Painting, drawing, printmaking, or sculpture	11.9%	5 (*very talented*)	12.6%
Gardening	12.2%	Self-rated effectiveness of activity at regulating emotions	
Reading novels, stories, poetry, or plays	12.3%	Not at all effective	3.0%
Playing a musical instrument	9.9%	Not very effective	5.8%
Cookery or baking	10.1%	A little effective	17.6%
Textile crafts such as embroidery, crocheting, or knitting	7.5%	Quite effective	48.7%
Creative writing	6.8%	Very effective	25.0%
Dancing	5.4%		
Photography	4.6%		
Composing music	1.6%		
Wood crafts such as carving or furniture making	1.5%		
Creating artworks or animations on a computer	1.3%		
Pottery, calligraphy, or jewelry making	1.0%		
Rehearsing or performing in a play/drama/opera/musical theater	.8%		
Making films or videos	.6%		
Learning or practicing magic tricks or circus skills	.2%		
*Note*. GCSE/CSE/O-levels = General Certificate of Secondary Education; *M* = mean; *SD* = standard deviation.
