# Supplementary material for: The Relationship Between Demographics, Behavioral and Experiential Engagement Factors, and the Use of Artistic Creative Activities to Regulate Emotions
Source: Psychol Aesthet Creat Arts. 2020 Jan 13;19(3):439–49. doi: 10.1037/aca0000296 (PMC12096857; doi:10.1037/aca0000296)
Supplement: Supplementary file 1 [file ACA-2018-0226_Suppl.docx]

Supplementary Table 1: Correlation matrix of continuous or ordinal variables included in the SEM

|  | Age | Education | Occupational status | Income | Experience | Training | Engagement frequency | Enjoyment | Talent | Success |
| --- | --- | --- | --- | --- | --- | --- | --- | --- | --- | --- |
| Age |  |  |  |  |  |  |  |  |  |  |
| Education | **-0.0885** |  |  |  |  |  |  |  |  |  |
| Occupational status | **0.2601** | **-0.142** |  |  |  |  |  |  |  |  |
| Income | **-0.0817** | **0.2082** | **-0.3534** |  |  |  |  |  |  |  |
| Experience | **0.3802** | 0.0024 | **0.0395** | 0.0042 |  |  |  |  |  |  |
| Training | **-0.0315** | **0.1391** | **0.0258** | **-0.0175** | **0.1393** |  |  |  |  |  |
| Engagement frequency | **0.0299** | -0.0018 | **0.055** | **-0.0372** | **0.1791** | **0.0719** |  |  |  |  |
| Enjoyment | **0.0527** | 0.0076 | **0.0523** | **-0.0615** | **0.1435** | **0.1239** | **0.315** |  |  |  |
| Talent | -0.0059 | **0.0613** | **0.0326** | **-0.0222** | **0.2136** | **0.2397** | **0.284** | **0.3373** |  |  |
| Success | 0.0041 | **0.0109** | **0.0318** | **-0.0591** | **0.0629** | **0.1447** | **0.2076** | **0.4522** | **0.2819** |  |

All values show Pearson’s correlation coefficient (r). Boldface indicates significance p<.05

Supplementary Table 2: Unstandardised and standardised beta coefficients and p values for SEM1

|  | B | β | | P(>\|z\|) | |  |
| --- | --- | --- | --- | --- | --- | --- |
| Latent variables: | | |  | |  | |
| SES=~ |  |  | |  | |  |
| Education | 0.216 | 0.239 | |  | |  |
| Income | 0.637 | 0.478 | | <.001 | |  |
| Employment | -0.789 | -0.739 | | <.001 | |  |
| Avoidance strategies=~ |  |  | |  | |  |
| q8 | 0.648 | 0.689 | |  | |  |
| q11 | 0.727 | 0.721 | | <.001 | |  |
| q15 | 0.691 | 0.72 | | <.001 | |  |
| q22 | 0.746 | 0.77 | | <.001 | |  |
| q23 | 0.739 | 0.823 | | <.001 | |  |
| q24 | 0.665 | 0.71 | | <.001 | |  |
| q43 | 0.766 | 0.782 | | <.001 | |  |
| Approach strategies=~ |  |  | |  | |  |
| q6 | 0.696 | 0.702 | |  | |  |
| q13 | 0.744 | 0.745 | | <.001 | |  |
| q28 | 0.754 | 0.777 | | <.001 | |  |
| q34 | 0.766 | 0.749 | | <.001 | |  |
| q44 | 0.776 | 0.786 | | <.001 | |  |
| q48 | 0.727 | 0.692 | | <.001 | |  |
| Self-development strategies=~ |  |  | |  | |  |
| q12 | 0.805 | 0.723 | |  | |  |
| q16 | 0.768 | 0.807 | | <.001 | |  |
| q31 | 0.749 | 0.813 | | <.001 | |  |
| q37 | 0.76 | 0.797 | | <.001 | |  |
| q40 | 0.679 | 0.681 | | <.001 | |  |
| factortot=~ | | |  | |  | |
| Avoidance strategies | 0.744 | 0.744 | |  | |  |
| Approach strategies | 0.863 | 0.863 | | <.001 | |  |
| Self-development strategies | 0.846 | 0.846 | | <.001 | |  |
|  |  |  | |  | |  |
| Regressions: | | |  | |  | |
| factortot~ | | |  | |  | |
| Age | -0.001 | -0.019 | | 0.001 | |  |
| Gender | 0.242 | 0.12 | | <.001 | |  |
| Ethnicity | 0.074 | 0.021 | | <.001 | |  |
| SES | -0.052 | -0.052 | | <.001 | |  |
| Training | 0.088 | 0.101 | | <.001 | |  |
| Experience | -0.019 | -0.026 | | <.001 | |  |
| Training~ |  |  | |  | |  |
| SES | -0.03 | -0.026 | | 0.001 | |  |
| Frequency of engagement~ |  |  | |  | |  |
| Open personality | 0.005 | 0.021 | | <.001 | |  |
| Enjoyment~ |  |  | |  | |  |
| Open personality | 0.007 | 0.048 | | <.001 | |  |
| Success at regulating emotions~ | | |  | |  | |
| Open personality | 0.017 | 0.085 | | <.001 | |  |
| Frequency of engagement~ |  |  | |  | |  |
| Perceived talent | 0.254 | 0.247 | | <.001 | |  |
| Enjoyment~ |  |  | |  | |  |
| Perceived talent | 0.128 | 0.223 | | <.001 | |  |
| Training~ |  |  | |  | |  |
| Age | -0.003 | -0.038 | | <.001 | |  |
| Experience~ |  |  | |  | |  |
| Age | 0.038 | 0.384 | | <.001 | |  |
| Frequency of engagement~ |  |  | |  | |  |
| Age | 0.001 | 0.019 | | <.001 | |  |
| SES~ |  |  | |  | |  |
| Age | -0.022 | -0.318 | | <.001 | |  |
|  |  |  | |  | |  |
| Covariances: | | |  | |  | |
| .factortot~~ | | |  | |  | |
| .Frequency of engagement | 0.096 | 0.091 | | <.001 | |  |
| .Enjoyment | 0.184 | 0.313 | | <.001 | |  |
| .Success at regulating emotions | 0.419 | 0.485 | | <.001 | |  |
| .Frequency of engagement~~ |  |  | |  | |  |
| .Enjoyment | 0.148 | 0.24 | | <.001 | |  |
| .Enjoyment~~ | | |  | |  | |
| .Success at regulating emotions | 0.193 | 0.378 | | <.001 | |  |
| .Frequency of engagement~~ |  |  | |  | |  |
| .Success at regulating emotions | 0.12 | 0.132 | | <.001 | |  |
| .Training~~ | | |  | |  | |
| .Experience | 0.249 | 0.165 | | <.001 | |  |
| Perceived talent | 0.29 | 0.239 | | <.001 | |  |
| .Experience~~ | | |  | |  | |
| Perceived talent | 0.317 | 0.232 | | <.001 | |  |

Supplementary Table 2: Unstandardised and standardised beta coefficients and p values for SEM2

|  | B | β | | P(>\|z\|) | |  |
| --- | --- | --- | --- | --- | --- | --- |
| Latent variables: | | |  | |  | |
| SES=~ |  |  | |  | |  |
| Education | 0.216 | 0.239 | |  | |  |
| Income | 0.637 | 0.478 | | <.001 | |  |
| Employment | -0.79 | -0.74 | | <.001 | |  |
| Avoidance strategies=~ |  |  | |  | |  |
| q8 | 0.647 | 0.689 | |  | |  |
| q11 | 0.726 | 0.72 | | <.001 | |  |
| q15 | 0.69 | 0.72 | | <.001 | |  |
| q22 | 0.744 | 0.768 | | <.001 | |  |
| q23 | 0.738 | 0.823 | | <.001 | |  |
| q24 | 0.665 | 0.71 | | <.001 | |  |
| q43 | 0.765 | 0.782 | | <.001 | |  |
| Approach strategies=~ |  |  | |  | |  |
| q6 | 0.696 | 0.701 | |  | |  |
| q13 | 0.746 | 0.746 | | <.001 | |  |
| q28 | 0.756 | 0.778 | | <.001 | |  |
| q34 | 0.768 | 0.75 | | <.001 | |  |
| q44 | 0.778 | 0.787 | | <.001 | |  |
| q48 | 0.729 | 0.693 | | <.001 | |  |
| Self-development strategies=~ |  |  | |  | |  |
| q12 | 0.806 | 0.724 | |  | |  |
| q16 | 0.764 | 0.805 | | <.001 | |  |
| q31 | 0.747 | 0.812 | | <.001 | |  |
| q37 | 0.758 | 0.795 | | <.001 | |  |
| q40 | 0.68 | 0.682 | | <.001 | |  |
|  |  |  | |  | |  |
| Regressions: | | |  | |  | |
| Avoidance strategies~ |  |  | |  | |  |
| Age | -0.002 | -0.027 | | <.001 | |  |
| Gender | 0.271 | 0.135 | | <.001 | |  |
| Ethnicity | -0.002 | -0.001 | | 0.913 | |  |
| SES | -0.015 | -0.015 | | 0.022 | |  |
| Training | 0.018 | 0.021 | | <.001 | |  |
| Experience | -0.003 | -0.004 | | 0.429 | |  |
| Approach strategies~ |  |  | |  | |  |
| Age | -0.002 | -0.032 | | <.001 | |  |
| Gender | 0.209 | 0.104 | | <.001 | |  |
| Ethnicity | 0.084 | 0.024 | | <.001 | |  |
| SES | -0.044 | -0.044 | | <.001 | |  |
| Training | 0.03 | 0.035 | | <.001 | |  |
| Experience | 0.008 | 0.012 | | 0.032 | |  |
| Self-development strategies~ |  |  | |  | |  |
| Age | 0.002 | 0.028 | | <.001 | |  |
| Gender | 0.124 | 0.062 | | <.001 | |  |
| Ethnicity | 0.042 | 0.012 | | 0.015 | |  |
| SES | -0.043 | -0.043 | | <.001 | |  |
| Training | 0.123 | 0.142 | | <.001 | |  |
| Experience | -0.057 | -0.081 | | <.001 | |  |
| Training~ |  |  | |  | |  |
| SES | -0.03 | -0.026 | | 0.001 | |  |
| Frequency of engagement~ |  |  | |  | |  |
| Open personality | 0.009 | 0.036 | | <.001 | |  |
| Enjoyment~ |  |  | |  | |  |
| Open personality | 0.014 | 0.102 | | <.001 | |  |
| Success at regulating emotions~ | | |  | |  | |
| Open personality | 0.034 | 0.169 | | <.001 | |  |
| Frequency of engagement~ |  |  | |  | |  |
| Perceived talent | 0.256 | 0.248 | | <.001 | |  |
| Enjoyment~ |  |  | |  | |  |
| Perceived talent | 0.123 | 0.214 | | <.001 | |  |
| Training~ |  |  | |  | |  |
| Age | -0.003 | -0.038 | | <.001 | |  |
| Experience~ |  |  | |  | |  |
| Age | 0.038 | 0.384 | | <.001 | |  |
| Frequency of engagement~ |  |  | |  | |  |
| Age | 0.001 | 0.019 | | <.001 | |  |
| SES~ |  |  | |  | |  |
| Age | -0.022 | -0.318 | | <.001 | |  |
|  |  |  | |  | |  |
| Covariances: | | |  | |  | |
| .Avoidance strategies~~ |  |  | |  | |  |
| .Frequency of engagement | 0.087 | 0.083 | | <.001 | |  |
| .Enjoyment | 0.165 | 0.28 | | <.001 | |  |
| .Success at regulating emotions | 0.322 | 0.373 | | <.001 | |  |
| .Approach strategies~~ |  |  | |  | |  |
| .Frequency of engagement | 0.074 | 0.071 | | <.001 | |  |
| .Enjoyment | 0.121 | 0.207 | | <.001 | |  |
| .Success at regulating emotions | 0.332 | 0.385 | | <.001 | |  |
| .Self-development strategies~~ |  |  | |  | |  |
| .Frequency of engagement | 0.072 | 0.069 | | <.001 | |  |
| .Enjoyment | 0.167 | 0.284 | | <.001 | |  |
| .Success at regulating emotions | 0.348 | 0.404 | | <.001 | |  |
| .Avoidance strategies~~ |  |  | |  | |  |
| .Approach strategies | 0.648 | 0.648 | | <.001 | |  |
| .Self-development strategies | 0.606 | 0.606 | | <.001 | |  |
| .Approach strategies~~ |  |  | |  | |  |
| .Self-development strategies | 0.742 | 0.742 | | <.001 | |  |
| .Avoidance strategies~~ |  |  | |  | |  |
| Open personality | 0.576 | 0.131 | | <.001 | |  |
| .Approach strategies~~ |  |  | |  | |  |
| Open personality | 0.745 | 0.17 | | <.001 | |  |
| .Self-development strategies~~ |  |  | |  | |  |
| Open personality | 0.976 | 0.223 | | <.001 | |  |
| .Frequency of engagement~~ |  |  | |  | |  |
| .Enjoyment | 0.147 | 0.239 | | <.001 | |  |
| .Enjoyment~~ | | |  | |  | |
| .Success at regulating emotions | 0.191 | 0.378 | | <.001 | |  |
| .Frequency of engagement~~ |  |  | |  | |  |
| .Success at regulating emotions | 0.118 | 0.131 | | <.001 | |  |
| .Training~~ | | |  | |  | |
| .Experience | 0.249 | 0.165 | | <.001 | |  |
| Perceived talent | 0.29 | 0.239 | | <.001 | |  |
| .Experience~~ | | |  | |  | |
| Perceived talent | 0.317 | 0.232 | | <.001 | |  |
